# Detection of myocardial enzymes, cardiac troponin T and hepatic and renal function in the diagnosis and treatment of severe pneumonia in children

**DOI:** 10.12669/pjms.345.15397

**Published:** 2018

**Authors:** Fengxia Han, Jianhong Gao, Jinghua Gai

**Affiliations:** 1 Fengxia Han, Clinical Laboratory, Binzhou People’s Hospital, Shandong 256610, China; 2 Jianhong Gao, Central Lab, Binzhou People’s Hospital, Shandong 256610, China; 3 Jinghua Gai, Medical Insurance Office, Binzhou People’s Hospital, Shandong 256610, China

**Keywords:** Hepatic and renal function and troponin T, Myocardial enzymes

## Abstract

**Objective:**

To analyze the significance of myocardial enzymes, cardiac troponin T (cTnT) and hepatic and renal function in the treatment of severe pneumonia in children.

**Methods:**

One hundred and twenty children with severe pneumonia who were admitted to the hospital between April 2015 and February 2017 were selected and included as a severe pneumonia group; 120 children with common pneumonia were included as a common pneumonia group; 100 healthy children were included as controls. The myocardial enzymes, cTnT and hepatic and renal function of patients in the three groups were detected and compared. The children with severe pneumonia were divided into a mild hypoxia group, a moderate hypoxia group and a severe hypoxia group according to arterial partial pressure of oxygen; the myocardial enzymes, hepatic and renal function and cTnT of the children in the three groups were compared. The correlations of partial pressure of blood oxygen with myocardial enzymes, hepatic and renal function and cTnT were analyzed.

**Results:**

The levels of myocardial enzymes, hepatic and renal function and cTnT of the severe pneumonia group, common pneumonia group and control group declined, and the differences had statistical significance (P<0.05). The levels of myocardial enzymes, hepatic and renal function and cTnT were higher in the children with severe hypoxia. The partial pressure of blood oxygen was in a negative correlation with myocardial enzymes, hepatic and renal function and cTnT in the severe pneumonia group.

**Conclusion:**

Timely monitoring of myocardial enzymes, hepatic and renal function and cTnT has an extremely important role in the evaluation of children with severe pneumonia.

## INTRODUCTION

Children have higher risks of severe pneumonia after being invaded by pathogenic bacteria because of the incomplete development of functional organs and immune system, insufficient respiratory mucosa secretory immunoglobulin A, weak cough and expectoration abilities and poor swallowing reflex. Up to this day, severe pneumonia remains to be a common disease in the pediatric field and one of the factors inducing death.^1^,^2^ An investigation suggested that about 14 million children under five years develop severe pneumonia every year around the world.^3^ Besides hypoxia, severe pneumonia, an infectious disease with high death rate, can induce severe complications such as cardiac failure, disseminated intravascular coagulation and severe sepsis and finally lead to death.^4^,^5^ Currently many indicators have certain references in reflecting the severity of severe pneumonia. Jain S et al. pointed out that procalcitonin (PCT) < 7 ng/mL could reflect a good prognosis and that high level of PCT usually indicated increased death rate.^6^ Usuda D et al. considered that monitoring level of brain natriuretic peptide could reflect the prognosis of patients and carrying out interventional treatment timely could enhance success rate of rescue.^7^ A study suggested that high level of D-dimer could reflect the severity of pneumonia and high risks of death.^8^ But these studies all focused on the changes of relevant indicators in the whole disease cycle, which cannot help make accurate prediction in the early stage. Timely and definite diagnosis and interventional treatment is of great significance in clinical treatment. It has been found that monitoring the dynamics of myocardial enzymes and cardiac troponin T (cTnT) has high values in evaluating the severity of severe pneumonia in children.^9^,^10^ In this study, the values of detection of myocardial enzymes, cTnT and hepatic and renal function in the treatment of severe pneumonia in children were analyzed.

## METHODS

One hundred and twenty children with severe pneumonia who were admitted to the hospital between April 2015 and February 2017 were selected and included as a severe pneumonia group. One hundred and twenty children with common pneumonia were included as a common pneumonia group. The severe pneumonia group included 70 males and 50 females; they aged from 6 months and 10 years (average 5.86±1.07 years); body mass was between 8.6 kg and 30.4 kg (average 23.89±1.13 kg). As regards symptoms, there were 71 cases of cough/expectoration and 49 cases of dyspnea. There were 54 cases of mild hypoxia, 50 cases of moderate hypoxia and 16 cases of severe hypoxia. The common pneumonia included 60 males and 40 females; they aged from five months and 10 years (average 5.83±1.06 years); the body mass of them was between 8.4 kg and 30.7 kg (average 23.92±1.11 kg). As to the symptoms, there were 74 cases of cough/expectoration and 56 cases of dyspnea. Patients who satisfied relevant diagnostic and degree criteria described in the Guidelines for the Diagnosis and Management of Community-Acquired Pneumonia edited by Respiratory Society of Chinese Medical Association^11^, were admitted to pediatric intensive care unit for at least 24 hours, could operate with treatment, could be tracked for outcome, and had complete clinical data were included. Those who had leukemia and systemic infection, had diseases such as bronchial asthma, bronchopulmonary dysphasia, foreign body aspiration and latent tuberculosis infection, had chronic cardiac insufficiency, had underwent anticoagulant therapy on admission, had history of primary blood coagulation disorder and congenital coagulation factor abnormality, or took glucocorticoids or immunosuppressive agents for a long term were excluded. One hundred healthy children were selected as controls. There were 59 males and 41 females; they aged from 7 months to 10 years (average 5.80±1.06 years). Body mass of the controls was between 8.2 kg and 30.5 kg (average 23.91±1.13 kg). No differences were observed in the general data between the three groups (P>0.05); therefore, the results were comparable. The study protocol was reviewed and approved by the ethics committee of the hospital, and the family members of the included children had signed informed consent.

### Methods

4 mL of fasting venous blood was collected from each patient who has fasted for more than 24 hour in the three groups. Myocardial enzymes and hepatic and renal function indexes including creatine kinase (CK), lactic dehydrogenase (LDH), α-hydroxybutyrate dehydrogenase (α-HBDH), aspartate transaminase (AST), creatine kinase-isozyme MB (CK-MB), urine creatinine (UCr) and alanine transaminase (ALT) were detected using a fully automatic biochemical analyzer (AU5800, Beckman Coulter, Inc., USA). Levels of cTnT and albumin (Alb) in the serum were detected using Biotin-Streptavidin enzyme-linked immunosorbent assay (BSA-ELISA), micro plate reader and kit (Beckman Coulter, Inc., USA). Arterial blood gas analysis was needed for the children with severe pneumonia. The level of arterial partial pressure of oxygen (PaO_2_) was detected to grade hypoxia; the level of PaO_2_ < 7.69 kPa was determined as mild hypoxia, the level of PaO_2_ between 5.29 kPa and 7.69 kPa was determined as moderate hypoxia, and the level of PaO_2_ < 5.29 kPa was determined as severe hypoxia. Firstly all the articles needed were prepared. Whether the injectors used were air-proof should be checked before blood collection. The whole needle tubing was immersed with a small amount of heparin; if no bubble appeared in the injector, then the injector was considered air-proof. 3 mL of blood was collected from the radial artery of each patient. The amount of the blood should not be excessive; otherwise anticoagulation results would be affected. The needle head was sealed, and the samples were mildly shaken. Finally the blood samples were sent for testing.

### Observation indicators

The levels of LDH, AST, α-HBDH, CK-MB, CK and cTnT and hepatic and renal function indicators (UCr, Alb and ALT) of the children in the three groups, the levels of cTnT and myocardial enzymes and hepatic and renal function indicators of the children with severe pneumonia, i.e. children in the mild, moderate and severe hypoxia groups, and the correlations of partial pressure of blood oxygen, myocardial enzymes, hepatic and renal function and cTnT were observed and analyzed.

### Statistical analysis

Data were analyzed using SPSS version 22.0. Measurement data normally distributed were expressed as mean ± standard deviation (SD) and compared using t-test. Enumeration data were expressed as percentage. Comparison between groups was carried out using Chi-square test. If P<0.05, then the difference was considered as statistically significant.

## RESULTS

The levels of myocardial enzymes and cTnT and the values of hepatic and renal function indicators were the highest in the severe pneumonia, followed by the common pneumonia group and control group (P<0.05, Table-I).

**Table-I:** Comparison of myocardial enzymes, cTnT and hepatic and renal function indicators between the severe pneumonia, common pneumonia and control groups (mean ± SD).

Group		Severe pneumonia group	Common pneumonia group	Control group	t	P
Myocardial enzymes	LDH(U/L)	287.41±51.26	195.42±49.33	200.33±2.35	8.552	<0.05
	AST(U/L)	75.62±8.41	41.27±7.42	25.27±1.11	8.328	<0.05
	Α-HBDH(U/L)	335.50±45.35	215.48±42.46	80.53±1.36	8.427	<0.05
	Creatine phosphate kinase(U/L)	262.26±41.63	149.48±37.51	45.88±1.12	8.433	<0.05
	Creatine phosphate kinase-MB(U/L)	42.36±4.57	21.63±4.28	12.09±1.10	8.342	<0.05
	cTnT(μg/L)	1.05±0.27	0.05±0.01	0.02±0.01	8.119	<0.05
Hepatic and renal functions	Serum creatinine (μmol/L)	110.37±1.43	88.46±1.45	40.32±1.38	8.311	<0.05
	Serum Alb(g/L)	93.41±4.13	60.24±3.26	27.34±1.08	8.343	<0.05
	ALT(U/L)	78.33±6.41	45.33±4.27	24.08±1.11	8.306	<0.05

The levels of myocardial enzymes, cTnT and hepatic and renal function indicators of the severe hypoxia group were significantly lower than those of the moderate hypoxia group (P<0.05), and the levels of myocardial enzymes, cTnT and hepatic and renal function indicators of the moderate hypoxia group were significantly lower than those of the mild hypoxia group (P<0.05, Table-II). The partial pressure of blood oxygen was in a negative correlation with myocardial enzymes, hepatic and renal function indicators and cTnT in the severe pneumonia group (p<0.05) (Table-III).

**Table-II:** Comparison of myocardial enzymes, cTnT and hepatic and renal function indicators between children with different severity of hypoxia (mean ± SD)

Group		Severe hypoxia group	Moderate hypoxia group	Mild hypoxia group	t	P
Myocardial enzymes	LDH(U/L)	254.93±15.57	307.67±16.02	227.42±15.55	10.876	<0.05
	AST(U/L)	96.52±3.64	73.46±4.61	49.59±3.22	9.655	<0.05
	Α-HBDH(U/L)	476.57±15.46	327.52±12.25	236.43±13.51	10.084	<0.05
	CK(U/L)	312.81±15.47	268.73±17.31	219.55±16.36	10.544	<0.05
	CK-MB(U/L)	65.13±3.26	43.13±3.03	25.72±2.27	11.016	<0.05
	cTnT(μg/L)	3.08±0.01	1.63±0.01	0.30±0.01	12.088	<0.05
Hepatic and renal functions	Serum creatinine(μmol/L)	117.83±0.70	104.61±0.77	95.12±0.63	9.644	<0.05
	Serum Alb(g/L)	112.46±4.50	87.52±3.24	69.66±3.55	9.349	<0.05
	ALT(U/L)	99.86±2.02	78.71±1.92	51.63±2.29	8.321	<0.05

**Table-III:** The correlations of partial pressure of blood oxygen with myocardial enzymes, hepatic and renal function indicators and cTnT in the severe pneumonia group.

Statistics	Myocardial enzymes					cTnT	Hepatic and renal functions		
	
	LDH	AST	α-HBDH	CK	CK-MB		Serum creatinine	Serum Alb	ALT
r	-0.879	-0.955	-0.948	-0.829	-0.979	-0.840	-0.912	-0.839	-0.819
P	<0.05	<0.05	<0.05	<0.05	<0.05	<0.05	<0.05	<0.05	<0.05

## DISCUSSION

Pneumonia is common among children as they have weak immunity. Infants under one year are of high risks to develop severe pneumonia.^12^ Myocardial damage is the most common complication of pneumonia, which is caused by the joint damage of enzymes and toxins generated by pathogens and many oxygen free radicals and inflammatory factors released by the body.^13^ Severe pneumonia accompanied with myocardial damage usually develops rapidly and can threaten lives in a short time. Therefore it is of great values to early diagnose severe pneumonia.

Myocardial cells have strong abilities of repairing and regeneration in the growth stage of children. Myocardial tissues can be repaired in a short time if myocardial damages are discovered and treated as soon as possible.^14^ But the symptoms and vital signs are not obvious in the early stage of pneumonia accompanied with myocardial damages. Moreover nearly no abnormal manifestations are displayed through conventional electrocardiograph examination. Currently whether a pneumonia patient has myocardial damage or not can be determined by detecting his myocardial enzymes and cTnT.

Myocardial cells contain many enzymes such as CK, LDH, α-HBDH, AST and CK-MB, which is called myocardial enzymes.^15^ LDH which has the highest level in the kidney, can be used for determining whether hepatic function is normal. The level of AST in the liver is secondary to that in the heart. When myocardial cells are threatened by inflammation and hypoxia, cell membrane permeability will increase immediately, and the aforementioned enzymes will generate automatically and enter blood to increase the release of CK.^16^ As CK, AST and LDH are not in myocardial cells and the specificities of myocardial injury are insufficient, only the increase of LDH and CK at the same time can indicate myocardial damage. LDH, LDH2 and CK-MB have high levels in myocardial cells, and α-HBDH can reflect the activity of LDH and LDH2; therefore α-HBDH and CK-MB are the specific indicators for myocardial damages in the early stage. When the activity of CK-MB exceeded 8%, its values in diagnosing myocardial damages are significant.^17^ A study has suggested that CK-MB had a specificity of 100% in the diagnosis of myocardial damages^18^, and its positive diagnosis rate was 96.45%. cTnT which can regulate myocardial contraction is usually in myocardial cells. Level of cTnT will increase several hours after the occurrence of myocardial damages. Level of cTnT is the highest in half a day or one day after damage, 40 or 50 times higher than the normal level, and will recover half a month later. Chen H et al. found that cTnT was specific to the damages of myocardial cells.^19^ Clinically manufactured monoclonal antibodies can specifically identify cTnT.

The results of the present study demonstrated that the levels of myocardial enzymes and cTnT and values of the hepatic and renal function indicators in the children with severe pneumonia were higher than those in the children with common pneumonia and controls. Compared to the healthy children, the above indicators had significant changes in both severe pneumonia and common pneumonia. The detection and analysis of the indicators will help initial determination of the category and severity of pneumonia. Moreover it was also found that the levels of cTnT and myocardial enzymes were significantly different between the children with mild, moderate and severe hypoxia in the severe pneumonia group; the severer the condition of hypoxia, the higher the level of cTnT and myocardial enzymes; the partial pressure of blood oxygen of the children with severe pneumonia was in a negative correlations with cTnT and myocardial enzymes. Therefore it could be concluded that the higher the levels of cTnT, hepatic and renal function indicators and myocardial enzymes in the children with severe pneumonia, the more serious the condition of myocardial damages.

### Limitations of the Study

The research time was short, and it was a single-center study with a small sample size; hence errors were inevitable. Therefore randomized controlled studies with larger sample size are needed to further verify the application values of those indicators.

## CONCLUSION

In conclusion, detection of myocardial enzymes, cTnT and hepatic and renal function is beneficial to the evaluation of disease progress and effect of clinical treatment in the treatment of severe pneumonia in children. As such it is preferable and worth promotion.

### Authors’ Contribution

**FXH:** Study design, data collection and analysis.

**JHG (Jianhong Gao) & JHG:** Manuscript preparation, drafting and revising.

**FXH & JHG (Jianhong Gao):** Review and final approval of manuscript.
